# Cardiovascular magnetic resonance in patients with mitral valve prolapse

**DOI:** 10.1016/j.jocmr.2024.101137

**Published:** 2024-12-25

**Authors:** Stefano Figliozzi, Silvana Di Maio, Georgios Georgiopoulos, Bert Vandenberk, Amedeo Chiribiri, Marco Francone, Nay Aung, Steffen E. Petersen, Tim Leiner, Jan Bogaert, Pier-Giorgio Masci

**Affiliations:** a IRCCS Humanitas Research Hospital, Milano, Italy; b Department of Biomedical Sciences, Humanitas University, Milano, Italy; c School of Biomedical Engineering and Imaging Sciences-Faculty of Life Sciences and Medicine, King's College London, London, UK; d Department of Clinical Therapeutics, National and Kapodistrian University of Athens, Athens, Greece; e Gasthuisberg University Hospital, Leuven, Belgium; f William Harvey Research Institute, Queen Mary University of London, London, UK; g Mayo Clinic, Rochester, Minnesota, USA

**Keywords:** Mitral valve prolapse, Mitral regurgitation, Sudden cardiac death

## Abstract

With a prevalence of 2–3% in the general population, mitral valve prolapse (MVP) is the most common valvular heart disease. The clinical course is benign in the majority of patients, although severe mitral regurgitation, heart failure, and sudden cardiac death affect a non-negligible subset of patients. Imaging of MVP was confined to echocardiography until a few years ago when it became apparent that cardiovascular magnetic resonance (CMR) could offer comparative advantages for detecting and quantifying mitral valve abnormalities alongside tissue myocardial characterization. The present review highlights the growing body of evidence supporting the role of CMR in patients with MVP. Based on the recent literature, CMR appears not as a simple alternative to echocardiography in patients with poor acoustic windows, but as a complementary imaging modality instrumental for better quantifying mitral valve abnormalities, mitral regurgitation severity, ventricular remodeling, and myocardial tissue changes. In this respect, pivotal CMR studies highlight that mitral annular disjunction and myocardial fibrosis by late gadolinium enhancement are associated with a heightened risk of life-threatening ventricular arrhythmias (arrhythmic MVP). We also delineate how these and other markers (e.g., the severity of mitral regurgitation) could enable a personalized risk assessment in patients with MVP and implement clinical decision-making. Here, we provide a comprehensive review of the current literature, with an emphasis on the arrhythmic MVP phenotype. The review also provides some practical suggestions on how to carry out a dedicated CMR protocol in MVP and composes a thorough report to inform clinicians on key aspects of this valvular heart disease.

## Graphical abstract

**Figure:**
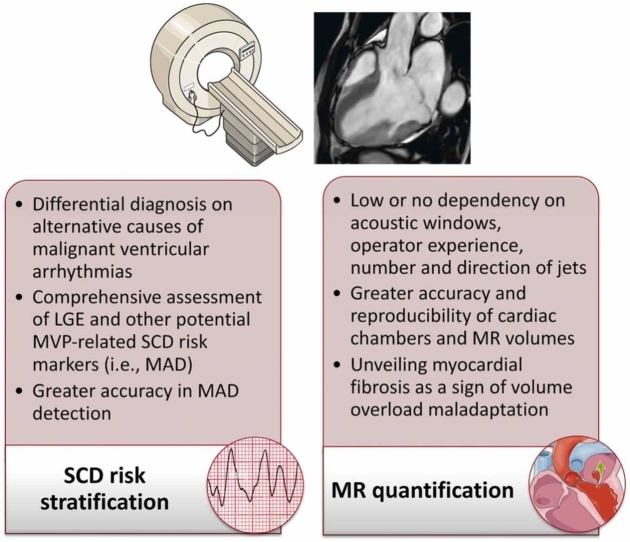
The added clinical role of CMR compared to echocardiography in patients with mitral valve prolapse. *CMR* cardiovascular magnetic resonance, *MAD* mitral annular disjunction, *MVP* mitral valve prolapse, *MR* mitral regurgitation, *LGE* late gadolinium enhancement, *SCD* sudden cardiac death

## 1 Introduction

### 1.1 An historical overview

The identification of patients affected by mitral valve prolapse (MVP) dates back to the inception of imaging in cardiology. In the 1960s, while the cardiology community in the Western Countries was mainly focused on rheumatic diseases, Dr. J.B. Barlow, a physician from South Africa, was intrigued by mid-systolic click associated with mid-late systolic apical murmur encountered in some young patients [1]. In one patient who died suddenly, he and his collaborators found “voluminous” mitral leaflets and elongated chordae tendineae at autopsy. Intriguingly, most patients with the above auscultatory findings had an exaggerated displacement of the mitral leaflets into the left atrium (LA) at the cine ventricular angiography, which was referred to as MVP. Thus, Barlow postulated that the mid-systolic click and mid-late systolic murmur at auscultation reflected mitral leaflets displacement into the LA and consequential mitral regurgitation (MR), respectively, as a result of myxomatous degeneration of the mitral apparatus. Barlow also noted that MVP was often co-segregated with connective tissue disorders, distinct electrocardiogram (ECG) patterns, ventricular arrhythmias, and proteiform symptoms configuring, in at least some patients, a clinical syndrome [1], [2].

Mitral annular disjunction (MAD), a morphological correlate of MVP, was described approximately 20 years later in a young victim of sudden cardiac death (SCD) with Barlow’s typical auscultatory pattern and myxomatous mitral valve degeneration. The investigators found an abnormal mitral annulus that appeared “elongated and the valve was moored on the left atrial side.” Compelling evidence supporting an association between MAD and MVP came a few years later. In a large sample of hearts from adult autopsies [3], the presence of MAD was found in 9 of 10 cases with a myxomatous degeneration of the mitral valve and only in 1 of 50 cases without such a feature. The authors postulated that the anatomical derangements of MAD accounted for greater mobility of the mitral valve in-vivo*,* encompassing the paradoxical expansion of the mitral annulus during systole, the so-called systolic curling [4], [5].

After 60 years of astounding technological progress, cutting-edge research, and clinical interest in MVP, we have to recognize Dr. J.B. Barlow for initial observational and conjectures based on a few dozen patients examined with limited resources, which laid the grounds for a deeper understanding of this fascinating disease.

## 2 Clinical appraisal

MVP is the most common valvular heart disease with a prevalence of 2–3% in the general population [6] and has a similar prevalence in men and women although bi-leaflet MVP is more prevalent in women [6], [7]. Early observational studies were affected by selection bias and reported 10–46% rate of cardiovascular complications in MVP patients, including heart failure, endocarditis, and SCD [8], [9], [10], [11]. In subsequent population-based studies, it became clear that the incidence of adverse cardiovascular events in MVP was much lower than initially described, ranging from 0.8–5.5% [6], [12]. Nevertheless, a subgroup of patients appeared to have an inherently high incidence of complications, including heart failure and SCD, at a rate of 112–408 events per 100,000 person-years [13].

From a morphological standpoint, MVP is appraised as a continuum of progressive degeneration of the mitral valve apparatus. Fibroelastic deficiency and Barlow's diseases sit at the opposite end. The former is typically characterized by thin leaflets, isolated prolapse in the P2 scallop, and limited mitral annulus enlargement. In contrast, Barlow’s phenotype features extensive myxomatous degeneration of both leaflets across multiple scallops, prominent annulus dilation, and lengthening of mitral chordae. The MVP can be *isolated* or *syndromic*, the latter being associated with systemic connective tissue disorders such as Marfan syndrome.

## 3 The role of cardiovascular magnetic resonance

### 3.1 Cardiovascular magnetic resonance in the diagnostic of MVP-MAD

**Fig. 1:**
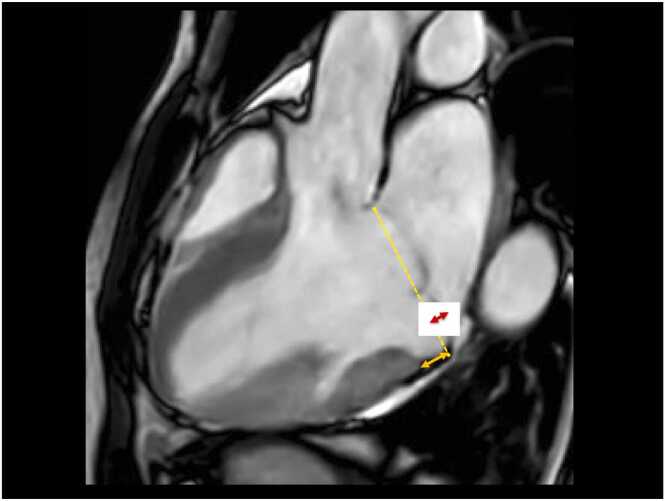
CMR diagnosis of mitral valve prolapse and mitral annular disjunction. Systolic frame of a standard three-chamber cine image of a patient with mitral valve prolapse (MVP) and mitral annular disjunction (MAD). The extent of MAD (continuous orange line), between the basal left ventricular and the mitral valve leaflet hinge-left atrial wall, should not be incorporated into the MVP quantification (continuous red line), which expresses the maximum atrial displacement of the mitral leaflets in systole. *CMR* cardiovascular magnetic resonance

The diagnosis of MVP by trans-thoracic echocardiography (TTE) or cardiovascular magnetic resonance (CMR) is defined by a systolic displacement of one or both mitral leaflets ≥2 mm above the anteroposterior plane of the mitral annulus in the parasternal long-axis view for TTE or in the three-chamber view for TTE and CMR [14] (Fig. 1).

The four-chamber and two-chamber views should not be used for this purpose, independently of the imaging modality used, because they image the ventricular-projected valleys of the saddle-shaped mitral annulus and, thus, give rise to a falsely exaggerated displacement of mitral leaflets into the LA [15], [16].

Accurate visualization of the mitral valve anatomy, the unparalleled ability to phenotype all cardiac chambers from the structural, geometrical, and functional standpoints, alongside the unique ability to non-invasively detect and quantify myocardial fibrosis (MF) render CMR an excellent imaging modality to delve into the complexity of MVP.

**Fig. 2:**
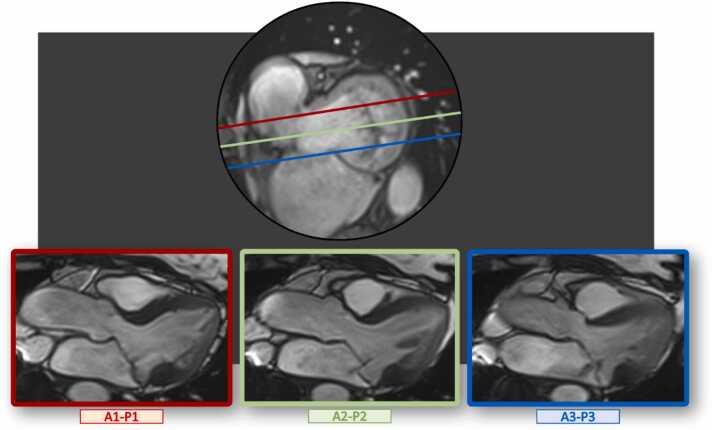
Comprehensive CMR characterization of mitral valve prolapse. To comprehensively characterize the mitral valve, a representative cine short-axis view at end-systole at the level of the mitral valve (upper panel) allows obtaining multiple cine three-chamber long-axis views (bottom panel) cross-cutting the three mitral scallops. *CMR* cardiovascular magnetic resonance

When TTE was used as the reference standard, Han et al. [17] showed that CMR cine imaging was 100% accurate in diagnosing MVP using TTE as the gold standard. Additional long-axis cine images parallel to the standard three-chamber view can be utilized to visualize structural and functional abnormalities along the A1-P1 and A3-P3 scallops to better probe the severity of prolapse (Fig. 2). Differently from TTE, the quality of cine imaging is not affected by body habitus, chest conformation, or lung diseases. However, cine imaging has lower temporal and spatial resolution than ultrasound. Thus, CMR may fall short in measuring mitral leaflet thickness or MVP-related complications such as flail due to chordal rupture or endocarditis, for which TTE and trans-esophageal-echocardiography (TOE) remain the imaging modalities of choice. The lower spatial and temporal resolution of CMR imaging are mitigated by the high contrast between the blood pool, mitral valve apparatus, and myocardium, which enables a thorough delineation of the structural and functional abnormalities of the mitral leaflets, *chordae*, papillary muscles, and mitral annulus. For instance, the high contrast between the posterior annulus-posterior leaflet and the basal inferolateral wall is instrumental in identifying and characterizing MAD in patients with MVP. In a cohort of consecutive patients with MVP, the prevalence of MAD was 17%, 26%, and 42% by TTE, TOE, and CMR, respectively, underscoring the higher sensitivity of cine CMR for diagnosing MAD compared to ultrasound [18].

### 3.2 Mitral Regurgitation

Severe MR causes left ventricular (LV) volumetric overload, pump failure, and eventually cardiac death. In MVP, multiple mechanisms contribute to MR, including leaflets malcoaptation, abnormal spatial relationship of the mitral apparatus components (particularly in adversely remodeled ventricle), and abnormal tethering of prolapsing leaflets. In some patients, MVP can trigger additional valvular abnormalities such as chordal rupture (particularly in elderly patients with fibroelastic deficiency) or endocarditis, further aggravating MR. The clinical indication for surgical repair relies largely upon the severity of MR [19], [20], [21]. Current guidelines recommend a labor-intensive operator-dependent approach based on a combination of qualitative, semi-quantitative, and quantitative echocardiographic parameters, which are often, not surprisingly, contradictory. Purely quantitative metrics, such as MR volume and effective regurgitant orifice area, have been advocated to better inform clinical decision-making. The proximal-isovelocity-surface-area (PISA) represents the most utilized and validated echocardiographic method to quantify MR volume and effective regurgitant orifice area. However, PISA is constrained by several physics principles (e.g., conservation of mass), operator-dependent maneuvers (e.g., defying the aliasing velocity of the color mapping), and inaccuracies in defining the actual shape of Doppler flow convergence zone which is often not hemispheric, as required to correctly measure MR volume, but rather asymmetric or semi-elliptic. Overall, these factors may lead to incorrect MR quantification by a factor of ∼2 associated with a high inter-observer and intra-study variability [22].

CMR enables the quantification of MR volume by two approaches, relying upon a correct application of phase-contrast velocity-encoded sequence [23], [24].

The *direct* CMR approach applies two-dimensional (2D) phase-contrast velocity-encoded imaging onto the mitral annulus to map out the velocity differences between the LV and LA during the cardiac cycle. This enables physicians to identify and quantify the regurgitant volume throughout the systolic images. While the quantification of MR severity directly on the mitral valve plane is appealing, this approach is hindered by several technical limitations, including through-plane motion (mitral annulus excursion is often exaggerated in MR) and the saddle shape of the mitral annulus, which may result in multiple regurgitant jets occurring in different planes. Moreover, identifying the appropriate velocity encoding value (VENC) can be difficult and time-consuming in high-velocity regurgitant jets, and the locally generated gradients by eddy currents at the level of the mitral leaflets can distort the phase-contrast generated velocity maps hampering the quantification of regurgitant volume [25], [26]. Although limitations related to multiple eccentric jets can be potentially overcome by four-dimensional (4D) phase-contrast velocity-encoded imaging, at the moment, there is still a scarcity of data on the application of this technique in the quantification of MR in MVP [27], [28], [29].

The *indirect* CMR approach derives MR regurgitant volume from the difference between the overall LV stroke volume measured from short-axis cine images and the forward aortic volume obtained by phase-contrast velocity encoding at the sino-tubular junction. The regurgitant fraction normalizes the MR regurgitant volume by the overall LV stroke volume, resulting in a robust metric of MR severity. Given its straightforwardness and robustness, this approach is largely adopted in clinical practice. However, caution should be paid to aortic regurgitation, which, if not correctly accounted for, leads to an underestimation of MR [26].

Using dedicated cine short-axis views, CMR can also determine the planimetry of the anatomic regurgitant orifice, which permits an alternative method for MR quantification with good agreement with invasive catheterization and PISA methods [30], [31].

According to a recent consensus document based on prognostic data, MR is classified as mild or moderate when the regurgitant fraction is <20%, 20–39%, and as severe when the regurgitant fraction and volume are ≥40% and >55–60 mL, respectively, [32], [33]. Several studies highlighted the greater precision and accuracy of the CMR *indirect* approach when compared to TTE for MR quantification. Among 103 patients with isolated MR (47% with MVP), the inter-observer agreement in grading MR as severe was 90% for the CMR *indirect* approach and only 61% for echocardiography, and less than one-fourth of patients with severe MR at echocardiography had severe regurgitation confirmed by CMR [34]. The reason why TTE tends to overestimate MR severity compared to CMR might be related to the fact that echocardiographic parameters, such as the size of the color jet, PISA, and vena contracta, rely on a single measurement in a systolic frame when the regurgitant rate is greatest, and do not capture MR along the entire systole, differently from CMR [35].

The MR severity quantified using the *indirect* CMR approach correlated strongly with LV end-diastolic volume reduction (r = 0.85; p < 0.0001) after mitral valve surgery, while no correlation was found between TTE-based MR severity and post-surgery ventricular remodeling. In a prospective cohort of 109 asymptomatic patients with moderate-to-severe MR at TTE, a regurgitant volume >55 mL or regurgitant fraction >40% by the CMR *indirect* approach predicted, in contrast to TTE-measures, symptoms development or indications for mitral surgery during follow-up [36].

Overall, the current evidence suggests that the *indirect CMR* approach performs better in quantifying MR when compared to TTE, allowing for better identification of patients who transition from the asymptomatic to symptomatic state or fulfill the criteria for mitral valve surgery [26], [37]. However, further studies are needed to expand the guideline-based recommendations that currently limit the use of CMR to patients with inadequate acoustic windows or when in patients with a discrepancy between TTE-based grading of MR and symptoms [38]. CMR is particularly attractive for MR quantification in patients in whom TTE falls short in quantitating MR severity of MR including multiple eccentric regurgitant jets or leaflet flail [39]. Similarly, CMR has the theoretical advantages in the quantification of residual MR following mitral valve repair or replacement. Haberka et al. [40] have demonstrated that CMR can reclassify the severity of paravalvular leak after mitral valve replacement, as assessed by TOE, in almost 50% of patients. However, prospective randomized trials or comparative effectiveness studies testing CMR versus echocardiography are lacking, and MR quantification following transcatheter mitral valve repair by CMR can be hindered by magnetic field inhomogeneities and susceptibility artifacts due to metallic devices (i.e., Mitra-clip [41]). Moreover, CMR is not suited for hemodynamically unstable patients and falls short in measuring pulmonary artery systolic pressure (PAPs) which is a key marker in stratifying patients with MVP and MR and timing mitral valve intervention. However, CMR [21] can complement the information of TTE in assessing patients with increased pulmonary artery pressure given the ability to estimate right heart chambers' dimensions and function [38]. CMR can also assess pulmonary artery flow by phase-contrast imaging, providing insights into pulmonary vascular and right arterio-ventricular coupling [42] (Table 1).

**Table 1:** Comparison between cardiovascular magnetic resonance and echocardiography in their clinical value for assessing patients with mitral valve prolapse.

	TTE/TOE	CMR	Notes
MVP diagnosis	+++	+++	
Availability	+++	+	
Low costs	+++	+	
*MR assessment*			
MR mechanisms	+++	+/++	CMR might be considered in patients with poor acoustic windows at TTE/TOE. However, small clefts, calcifications, vegetations, and flails can be easily missed by CMR.
Suspected endocarditis	++/+++	+	Small valvular lesions can be easily missed by CMR. For the evaluation of peri-valvular complications, computed tomography/positron emission tomography is more accurate than CMR.
MR quantification	+/++	+++	CMR quantification has shown higher reproducibility and is not affected by the number and direction of regurgitant jets. TTE/TOE is prone to MR overestimation in patients with MVP. Physicians should adopt caution on CMR results in patients with atrial fibrillation, aortic stenosis, or cardiac devices.
Pulmonary artery systolic pressure estimation	+++	−/+	
Cardiac chambers’ dimensions and pump function	++	+++	Third-chamber volume should be excluded by left ventricular end-systolic volume. Accuracy of ventricular and atrial volumes significantly improves by using 3D-echocardiography compared to 2D-echocardiography.
Prolapsing “third chamber” dimension	+	+++	The added clinical value of this parameter has to be established (i.e., mainly confined to research purposes).
Myocardial fibrosis	−	+++	Myocardial fibrosis unveiled by alterations on T1-mapping techniques but not on LGE might reveal interstitial fibrosis that can regress after mitral valve repair/replacement.
Planning for mitral valve repair/replacement	+++	−/+	
Guidance for mitral valve repair/replacement	+++	−	
Follow-up after mitral valve repair/replacement	++	+	
*Arrhythmic risk assessment*			
Mitral annular disjunction	++	+++	CMR has demonstrated higher sensitivity and accuracy for this imaging finding, but clinical significance remains controversial.
Apical papillary muscles insertion	+	++	The added clinical value needs to be further established (i.e., mainly confined to research purposes).
Myocardial fibrosis	−	+++	LGE is supported by stronger clinical evidence than multiparametric mapping techniques. LGE of the papillary muscles might be challenging to assess.
Cardiac chambers’ dimensions and pump function	++	+++	Third-chamber volume should be excluded by left ventricular end-systolic volume. Accuracy of ventricular and atrial volumes significantly improves by using 3D-echocardiography compared to 2D-echocardiography.
Deformation imaging	++/+++	++	The added clinical value has to be established (i.e., mainly confined to research purposes).
Alternative diagnoses related to ventricular arrhythmias	+	+++	CMR acquisition protocols should be tailored based on patients’ clinical questions.

− Absent, −/+ doubtful, + weak, ++ moderate, +++ strong, *2D* two-dimensional, *3D* three-dimensional, *CMR* cardiovascular magnetic resonance, *LGE* late gadolinium enhancement, *LV* left ventricle, *MR* mitral regurgitation, *MVP* mitral valve prolapse, *TOE* trans-esophageal echocardiography, *TTE* trans-thoracic echocardiography

#### 3.2.1 Cardiac remodeling

Ventricular dilatation and dysfunction in MVP may reflect MR-mediated chronic volume overload, myocardium-intrinsic (cardiomyopathy-like) mechanisms, or both. CMR is the gold standard for the assessment of LV and LA volumes [38]. However, 3D-TTE, which is commonly used in many labs today, allows for much greater accuracy than conventional 2D-TTE in left heart volumes [43]. Interestingly, left heart chamber dilatation has been reported in patients with MVP, particularly in those with Barlow’s phenotype, which appears excessive and disproportionate to the degree of MR [44], [45]. This has generated speculation about two potential mechanisms underlying LV remodeling in MVP: (1) the “third-chamber” effect and (2) intrinsic myocardial abnormalities.

**Fig. 3:**
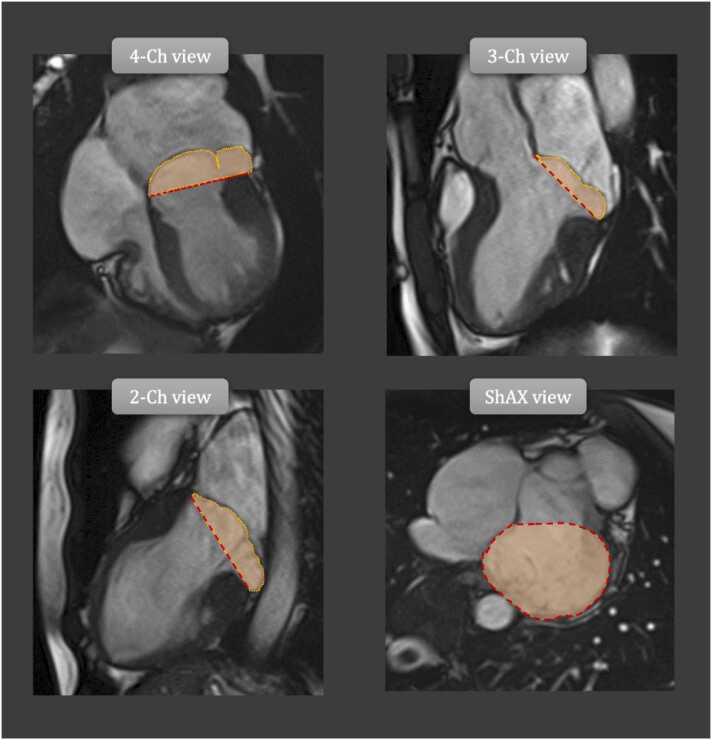
“Third-chamber” effect: a functional intraventricular regurgitation. Long-axis (4-Ch, 3-Ch, and 2-Ch) and a representative basal short-axis (ShAX) views encompassing the “third-chamber” or “prolapse volume.” This entity is defined as the volume comprised between the prolapsing mitral leaflets and the most basal part of the left ventricle. *2-Ch* two chamber, *3-Ch* three chamber, *4-Ch* four chamber

The former is particularly relevant in Barlow’s disease, where large prolapsing leaflets and a dilated mitral annulus concur to generate an end-systolic chamber (“third-chamber” or “prolapse volume”) (Fig. 3). This brings about a functional intraventricular regurgitation, which can impose substantial volume overload in addition to transmitral regurgitation [46]. Similarly, LA dilatation, which virtually always accompanies severe MR, has also been found in MVP patients with no more than mild MR [45]. In another study including 253 patients with MVP and 344 age- and sex-matched healthy controls with less than moderate MR, those with MVP showed larger LV and LA volumes than controls [46]. Notably, MR severity was not associated with left chamber dilation. However, the third-chamber effect was not investigated, preventing any conclusive remarks. Of interest, in a study including 157 patients with MVP, those with bi-leaflet MVP, and therefore larger “third-chamber,” had larger LV end-diastolic volumes compared to patients with single-leaflet MVP (smaller “third-chamber”) despite similar transmitral MR. Of note, the third-chamber functional regurgitation helped to better explain LV dilatation when added to the statistical model accounting only for transmitral regurgitant volume [46], [47]. These results argue against the presence of an underlying independent cardiomyopathy leading to LV dilation. Accordingly, an increase in right ventricular dimensions has not been demonstrated in patients with MVP [47], [48].

Evidences supporting intrinsic myocardial abnormalities include histological proof of interstitial fibrosis, mitochondrial degeneration, nuclear chromatin clumping, and myocyte degeneration in MVP patients, irrespective of MR [49], [50]. Given the strong inheritance of MVP, the histopathological evidence supports the knowledge that left-heart chamber dilatation and remodeling may represent an early phenotypic expression of a genetically mediated condition. This intriguing hypothesis is further substantiated by a recent meta-analysis of two genome-wide-association-studies, including nearly 1500 non-syndromic MVP cases, which identified and successfully independently replicated six loci, providing for the first time functional evidence for candidate genes likely involved in cytoskeleton organization [51], [52].

#### 3.2.2 Myocardial fibrosis

**Fig. 4:**
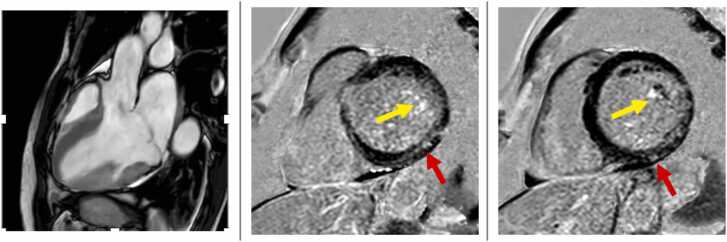
Myocardial fibrosis by late gadolinium enhancement (LGE) in a patient with mitral valve prolapse. Bi-leaflet mitral valve prolapse is evident in a systolic frame of three-chamber cine imaging (left). Typical LGE of the papillary muscles (yellow arrows) and the posterior left ventricular wall with a mid-wall pattern (red arrows) is evident in basal (mid) and mid (right) short-axis images

CMR has the unparalleled ability to non-invasively interrogate myocardial tissue composition by leveraging late gadolinium enhancement (LGE) (Fig. 4) and parametric mapping techniques. While the former enables the detection and quantification of replacement (scar-like) MF, pre/post-T1-mapping is instrumental in estimating extracellular volume fraction (ECV) by incorporating in a simple formula the patient’s hematocrit, native (pre-contrast), and post-contrast T1 relaxation time of myocardium at (pseudo) equilibrium. This biomarker enables non-invasively gauging the extracellular extravascular space of the myocardium, which reflects interstitial (diffuse) fibrosis in the absence of other conditions expanding the cardiac interstitium (e.g., amyloid deposition or edema) [53].

Over the past decade, mounting evidence from several observational CMR studies has shown a relatively high prevalence of MF in patients with MVP. In a cohort of patients with organic MR, those with MVP showed nearly four times higher prevalence of MF by LGE than patients with other forms of organic MR [54]. The MF is preferentially localized in the lateral wall, particularly in the region of the insertion of the posteromedial papillary muscle (generally referred to as the “peri-mitral myocardium”), and MF is associated with more advanced mitral apparatus derangements such as bi-leaflet prolapse, annulus disjunction, or MVP severity [55], [56]. This is in keeping with the hypothesis that more severe forms of MVP may prompt higher mechanical stress in the papillary muscles and adjacent wall, activating pro-fibrotic mechanisms through tension-mediated molecular pathways [5], [57]. Morningstar et al. [58] lent credence to this hypothesis by showing an enhanced mechano-sensing of cardiac fibroblasts, eventually translating into transforming growth factor beta gene expression and MF by computational modeling.

Kitkungvan et al. [54] studied 356 patients with MR (moderate or greater in 56% of patients) and reported a higher prevalence of MF by LGE in patients with MVP than other forms of MR (37% vs 7%; p < 0.001). The MF prevalence increased in parallel with MR severity being detected in only 10% of patients with less than mild MR (regurgitant fraction <10%) and up to 50% of patients with severe MR (regurgitant fraction ≥50%). Of note, nine patients had sustained ventricular tachycardia or SCD over a median 3.7-year follow-up, and more than half of these events occurred in patients with MVP and MF by LGE. Given the paucity of events, however, the predictive value of MF by LGE was not adjusted for other relevant covariates, such as MR severity, which incidentally was more severe in LGE-positive (regurgitant fraction of 41%) than LGE-negative patients (regurgitant fraction of 29%, p < 0.001).

In a retrospective observational two-center study including 400 patients with MVP and MR (moderate or greater in 70%), MF by LGE and MR severity were independently associated with cardiovascular death, worsening heart failure, *de-novo* atrial fibrillation, ventricular arrhythmias, or stroke over a median follow-up of 8.4 months. Of relevance, the clinical outcome was mainly driven by worsening heart failure symptoms in nearly two-thirds of cases, likely reflecting the nature of the study population (i.e., high prevalence of hemodynamically significant MR [59]).

It is worth noting that these studies fell short in elucidating the prognostic significance of MF by LGE in MVP, given that they included patients with a high prevalence of moderate-to-severe MR, which has been validated as a strong independent adverse prognosticator by large prospective population-based studies [60], [61], [62]. Increased ECV values have also been associated with symptoms’ onset and clinical decompensation [63], [64]. Unlike LGE, an increased ECV showed a similar fashion in MVP vs non-MVP cohorts with MR. Thus, LGE might more specifically reflect the pathophysiological abnormalities of MVP, whereas increased ECV better reflects volume overload regardless of the etiology.

### 3.3 The special case of arrhythmogenic MVP

Significant MR and LV systolic dysfunction have been associated with adverse outcomes, including a higher incidence of SCD [60], [62]. However, only one-fifth of SCD cases occur in MVP patients with severe MR [13]. Thus, most SCD cases in MVP are likely triggered by mechanisms other than MR or LV dysfunction, a phenotype referred to as arrhythmic MVP (a-MVP). Although the definition of a-MVP is somewhat arbitrary [65], prior studies showed that these patients are predominantly females with a median age of 30, and they often present with T-wave inversion/ST-segment depression in the inferolateral leads and frequent ventricular ectopy with polymorphic/right bundle-branch-block morphology [5], [12]. Patients with Barlow’s phenotype and severe bi-leaflet prolapse are traditionally considered at higher risk of SCD as compared to those with fibroelastic deficiency phenotype or mild single-leaflet prolapse.

**Fig. 5:**
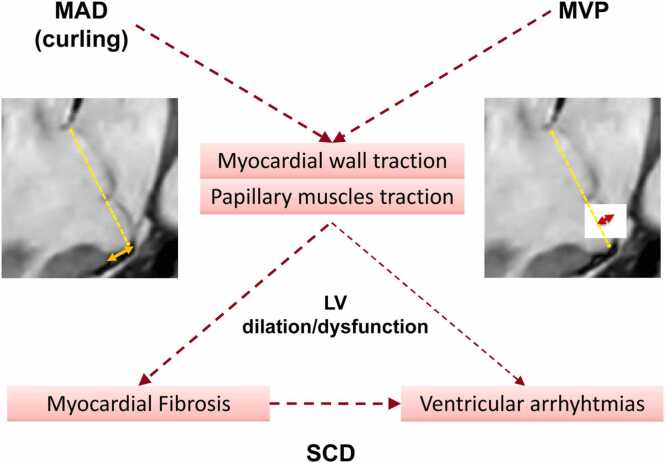
Proposed mechanisms underlying sudden cardiac death in patients with mitral valve prolapse. *MAD* mitral annular disjunction (continuous orange line), *MVP* mitral valve prolapse (continuous red line), *LV* left ventricle, *SCD* sudden cardiac death

Recently, MAD has gathered attention as another arrhythmogenic marker [66]. This entity reflects an abnormality of the mitral annulus, which normally retains a cordon-like shape in its posterior portion and, by contrast, acquires a curtain-like shape in annulus disjunction due to a looser assembly of collagen fibers [3], [5]. The functional consequence of MAD is an exaggerated apical-inward motion of the basal inferolateral LV wall (“curling”), which can be associated with abnormal annular expansion [5] (Fig. 1; Supplementary Video). This has been regarded as a potential mechanism of enhanced stretch at the insertion of papillary muscles in the lateral wall, promoting the onset of ventricular arrhythmias [5] (Fig. 5). As such, MAD caught the attention of several investigators, who reported an association between this anatomical landmark and complex ventricular arrhythmias [55], [66], [67], [68]. In 595 patients with MVP, Essayagh et al. showed that MAD at echocardiography was independently associated with severe ventricular arrhythmias [68]. The authors included relatively old patients (mean age of 65) with a high prevalence of comorbidities, and, more importantly, MR was moderate-to-severe in more than half of the cases. However, another study could not find any association between MAD and increased mortality at 10 years follow-up [69]. Three recent imaging studies reported a high prevalence of MAD in consecutive healthy subjects with no MVP or ventricular arrhythmia [70], [71], questioning the significance of MAD as a pro-arrhythmic marker. It must, however, be acknowledged that in these studies, the longitudinal (around 3 mm) and circumferential (mainly involving P1- and P3-scallops) extent of MAD was mild. This imaging entity was less frequently appreciated in the three-chamber view and likely reflects a normal morphological variant of posterior mitral annulus diverging from the form of annular disjunction observed in patients with MVP. In contrast, inferolateral MAD is mostly found in patients with MVP [70], [71], [72]. In a large cohort of consecutive patients undergoing CMR, only MADs of greater longitudinal extent (i.e., ≥4–6 mm) were associated with the burden of ventricular arrhythmias [72]. As a matter of fact, autopsy and imaging studies concurred in showing a close correlation between MVP severity and MAD, reporting MAD prevalence of only 9% in patients with single-leaflet mild prolapse and 98% in patients with severe Barlow’s bi-leaflet prolapse [34], [73]. Given that MAD co-segregates with MVP severity and both are associated with MF by LGE, separating out the contribution of these intertwined factors on clinical outcomes remains challenging. Faletra et al. have recently proposed a distinction between pseudo-MAD, present only in systole and secondary to a juxtaposition of the belly of the billowing posterior leaflet on the adjacent left atrial wall, and true-MAD, where the insertion of the posterior leaflet is clearly visualized in both diastole and systole [74]. However, data on the prevalence of pseudo-MAD and true-MAD and their clinical implications remain unknown. Notably, mitral valve surgery eliminates MAD by suturing the posterior ring and valve prosthesis mitigating MAD-related traction forces. Essayagh et al. found a trend toward a lower incidence of ventricular arrhythmias after surgery in patients with MAD/MVP [68].

Supplementary material related to this article can be found online at doi:10.1016/j.jocmr.2024.101137.

The following is the Supplementary material related to this article Video S1.

Video S1 **Supplementary Video 1.** Cine**-**imaging of a standard three-chamber cine image of a patient with mitral valve prolapse and mitral annular disjunction associated with systolic curling.

Studies investigating myocardial deformation by CMR in MVP are limited. Feature-tracking enabled to identify that reduced regional peak radial and circumferential strains in the LV lateral wall were associated with MF by LGE and compounded an increased likelihood of high ventricular arrhythmias burden (>5%) and ventricular tachycardia at Holter monitoring, or aborted SCD [75].

Arguably, MF is a key feature of a-MVP. In an autopsy series, Basso et al. reported that 100% and 88% of young adults with MVP without significant MR who died suddenly had MF in the papillary muscles and LV inferolateral wall, respectively [56]. The same group reported similar findings in living patients with MVP using LGE-CMR, reiterating the association between MF by LGE and ventricular arrhythmias [55], [56]. In a multicenter retrospective observational study including 474 patients with MVP without LV dysfunction or significant MR, Figliozzi et al. found MF by LGE in 6% of patients presenting with aborted SCD, whereas the prevalence of MF was only 1% (10-fold lower) in patients without such malignant presentation. Moreover, MF conveyed a four-fold increased risk of SCD, sustained ventricular tachycardia, and unexplained syncope over a median follow-up of nearly 4 years after correction for the major confounders. This result was confirmed when MF extent (% of LV mass) replaced the presence of MF by LGE in the multivariable models, further corroborating the importance of MF in an MVP and providing biological plausibility for this biomarker. The authors did not find an association between MAD and clinical outcomes in the same study. In an exploratory study, patients with MVP and complex ventricular arrhythmias showed shorter post-contrast T1 times when compared with patients with MVP without complex ventricular arrhythmias [76]. In a study including 30 patients with MVP, MF by LGE and increased ECV calculated in the six basal myocardial segments (>33.5%) performed equally well in identifying patients with a history of aborted SCD, [77] and increased ECV was more strongly associated with complex ventricular arrhythmias at Holter monitoring (class 4 or 5 of Lown) than MF by LGE. However, the measurement of ECV in the LV lateral wall by T1-mapping remains challenging, given the limited spatial resolution of single-shot source images and motion-prone artifacts occurring in this heart region. By assuming an actual increase in ECV, the underlying mechanisms of such abnormalities remain to be fully elucidated. For instance, a recent intriguing report studying 20 patients with MVP with 18F-fluorodeoxyglucose positron-emission-tomography reported that myocardial inflammation colocalizes with MF by LGE in 70% of the cases [78].

**Fig. 6:**
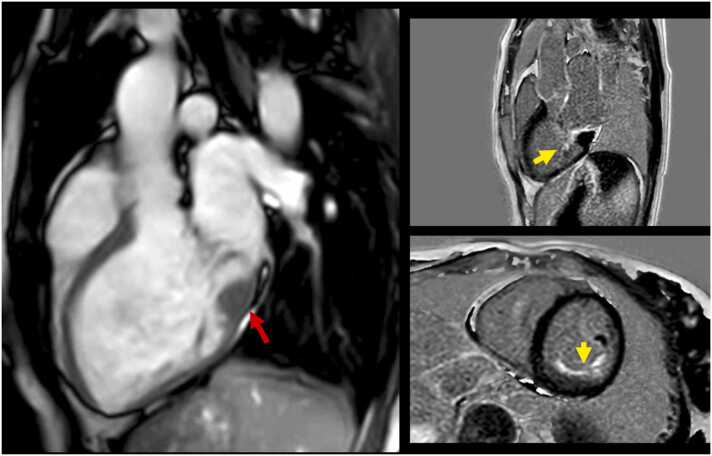
Relative hypertrophy of the basal posterior left ventricular wall in a patient with mitral valve prolapse. Relative hypertrophy of the basal posterior wall (red arrow; right) and late gadolinium enhancement of the papillary muscles (yellow arrows, left) in a patient with mitral valve prolapse (Barlow’s disease)

**Fig. 7:**
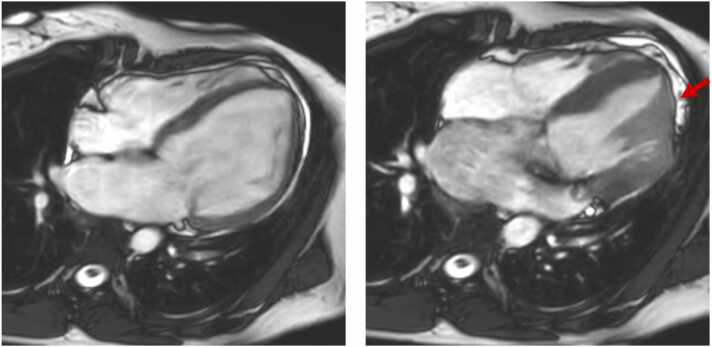
Apical papillary muscles insertion in a patient with mitral valve prolapse. Four-chamber cine imaging of a diastolic (left) and systolic (right) frame of a patient with mitral valve prolapse (Barlow’s disease). Apical papillary muscles insertion is evident (red arrow)

Recently, two additional structural abnormalities have also been reported in MVP: (1) a relative hypertrophy of the basal posterior LV wall [55] (Fig. 6), and (2) an apical papillary muscles insertion [79] (Fig. 7). The former finding likely reflects a localized remodeling secondary to high traction forces locally exerted by severe MVP/MAD [55]. Patients with apical papillary muscle insertion might be characterized by a higher tensile force on the myocardium than patients with normally implanted papillary muscles, resulting in a higher prevalence of LGE and a higher burden of ventricular arrhythmias [79].

## 4 Proposed CMR protocols to assess patients with MVP in daily practice

**Fig. 8:**
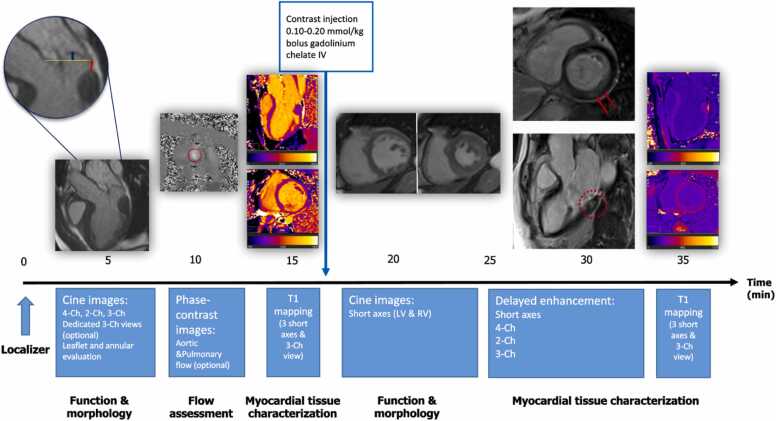
Proposed CMR protocol in patients with mitral valve prolapse. From left to right: after localizer sequences, long-axis cine images are acquired. The assessment of mitral valve prolapse (blue arrow) and mitral annular disjunction (red line) is performed in standard three-chamber cine images. Additional three-chamber cine stacks (i.e., 5 mm with no slice gap) are highly encouraged to comprehensively evaluate the mitral valve apparatus, including mitral regurgitation jet origins, directions, and prolapses in multiple scallops. Phase-contrast imaging should be used to quantify mitral regurgitation. In experienced centers, 4D flow is an alternative to standard 2D-phase-contrast velocity-encoded imaging. Pre-contrast T1-mapping evaluation should include basal- and mid-short-axis views and three-chamber views. Short-axis cinestacks of the ventricles are acquired for measuring bi-ventricular dimensions and functions. Late sequences acquired after contrast administration are crucial to characterize the presence and extent of myocardial fibrosis by late gadolinium enhancement by replicating the orientation of the long-axis and short-axis cine images. The typical pattern of late gadolinium enhancement in patients with mitral valve prolapse is mid-wall at basal-mid posterior segments (red circle and arrows). Finally, post-contrast T1-mapping techniques replicate the acquisition parameters of native sequences to calculate extracellular volumes. *2D* two-dimensional, *4D* four-dimensional, *CMR* cardiovascular magnetic resonance

The main features of a CMR protocol for MVP are summarized in Fig. 8.

*Phenotyping of the mitral valve apparatus:* First, a dedicated high spatial and temporal resolution stack of short-axis cine images should be planned at the level of the atrioventricular plane, fully encompassing the mitral valve annulus and leaflets. A trade-off between temporal and spatial resolution should be thoughtfully sought. A stack of three-chamber cine images with similar temporal and spatial resolution should be planned in the long-axis direction to fully encompass the mitral valve scallops (Fig. 2). Both stacks are instrumental for thoroughly characterizing the structural and functional abnormalities of the mitral annulus and leaflets, including MVP, MAD, and MR jets, which are not infrequently multiple with variable directions. By cross-relating the images throughout the cardiac cycle in the two orientations, MVP and related complications can be identified and quantified. It is not unusual that small flails and highly eccentric MR overlooked by prior investigations are singled out by this approach. Moreover, this approach enables us to gauge the severity of the prolapsing leaflets and MAD along the longitudinal (base-to-apex) and circumferential (mediolateral across the mitral scallops) directions. Based on evidence suggesting the association with ventricular arrhythmias [55], [67], [72], we propose measuring MAD in the three-chamber cine-view at the systolic frame showing maximum longitudinal extent.

*Chamber remodeling and ventricular systolic function*: Ventricular volumes, mass, systolic function, and atrial size are quantified by customary short-axis and long-axis cine images [80]. However, a few considerations should be taken into account. First, quantifying functional intraventricular regurgitation, the so-called “third-chamber,” would benefit from contiguous (no image gap) short-axis images with 5–6 mm slice thickness and no interslice gap. Thus, the customary stack of short-axis cine images should be adapted to implement the z-axis spatial resolution to quantify the “third-chamber” volume.

The LA is often dilated in MVP, also in the absence of significant MR. However, atrial size quantification using planimetry on the four-chamber long-axis cine is limited [81], and therefore, volumetric quantification by a stack of short-axis cine images should be considered.

*Mitral regurgitation quantification*: This can be easily achieved by applying the indirect approach outlined above by measuring the flow in the ascending aorta using 2D-phase-contrast velocity-encoded imaging at the level of the sino-tubular junction and LV stroke volume using the cine short-axis images stack. It would be sensible to acquire an additional 2D-phase-contrast velocity-encoded imaging at the level of the pulmonary artery as an internal control. Artifacts secondary to turbulent MR jets and high flow velocity may impair image quality and hinder exam interpretation. Using lower field strength (i.e., 1.5T instead of 3T), optimizing the magnetic field shimming and tailoring VENC can mitigate the artifacts resulting in better image quality and diagnostic yield. Electronic devices (i.e., pacemaker, implantable cardioverter defibrillator) are associated with susceptibility artifacts potentially leading to an underestimation of flow near to the device, and in this case raising the ipsilateral arm is a simple but effective approach to distance the device from the heart and mitigate artifacts. In experienced centers, 4D-phase-contrast velocity-encoded imaging can be an alternative to the conventional CMR *indirect* method to quantify MR, which is particularly attractive in patients with multiple and mixed heart valve diseases given the intrinsic limits of 2D-phase-contrast velocity-encoded imaging in these cases [24]. In the presence of multiple MR jets with different directions, which are not uncommon in patients with MVP, it is advisable to use the *indirect* 4D-phase-contrast velocity-encoded imaging method with valve tracking through the mitral and aortic valves, as this has been shown to be more accurate than *direct* 4D-phase-contrast velocity-encoded imaging tracking the mitral valve [82]. However, large-scale studies evaluating 2D vs 4D-phase-contrast velocity-encoded imaging in patients with MVP are awaited before widespread clinical implementation of the latter method.

*Tissue characterization*: The standard LGE technique represents the cornerstone of tissue characterization and should be carried out with high standards in MVP patients by replicating the orientation and spatial resolution of the long-axis and short-axis images. The presence of LGE should be confirmed by cross-cut images, phase swapping, or both. Subendocardial foci of fibrosis can be found in patients with MVP at the mid-ventricular lateral wall (most often at the insertion of the posteromedial papillary muscle). Dark-blood or black-blood LGE approaches should be considered to implement the visualization of this pattern of MF, which could be otherwise overlooked by conventional bright-blood LGE, particularly in case of similar signal intensity between the scar and the LV blood pool [80]. Tri-dimensional LGE acquisition might be considered for research when dealing with electroanatomic integration and searching for arrhythmic substrates, as shown in other diseases [83]. Similarly, pre/post T1-mapping and T2 mapping in short-axis or long-axis orientation are optional and mostly reserved for research.

*What to include in the report:* The description of mitral leaflets can include their length and thickness as measured on cine images. In Barlow’s MVP, leaflets are elongated and thickened and are often associated with sub-valvular abnormalities, including thickened chordae and apical insertion of the papillary muscles [84]. These structural abnormalities are much less pronounced or absent in fibroelastic deficiency. Subsequently, any functional abnormalities should be carefully detailed with respect to single or bi-leaflet prolapse and maximal longitudinal displacement of the leaflet(s) into the LA and the circumferential extent of the prolapse (scallop involvement) using the dedicated mitral valve long-axis and short-axis cine images, respectively. If present, the longitudinal extent of the MAD can be measured. The presence and degree of MR are usually reported by combining the information of the dedicated mitral valve cine images (number of jets and their direction, scallops involved) and the CMR *indirect* approach. Finally, the presence, location, and pattern of MF by LGE can be described qualitatively. Quantification and reporting of LGE and native T1-mapping abnormalities remain primarily confined to research settings, as their inclusion in clinical reports is not yet recommended. The main CMR parameters to be included in the report are synthesized in Table 2.

**Table 2:** Proposed parameters to include in the CMR report of patients with mitral valve prolapse.

	Acquisition	Analysis	Report
Cardiac chambers	Bi-ventricular and atrial cine imaging according to current guidelines [80].	Bi-ventricular and atrial volumes and ejection fraction according to current guidelines. Strain imaging is optional and mainly indicated for research purposes [80].	Bi-ventricular and atrial volumes and ejection fraction according to current guidelines [80]. Third-chamber volume should be excluded from the LV end-systolic volume because it would lead to an underestimated LV ejection fraction. Strain imaging is optional and mainly indicated for research purposes.
Mitral valve apparatus - anatomy	A stack of three-chamber cine images with high temporal and spatial resolution to fully encompass the mitral valve scallops.	MVP: maximum systolic atrial displacement in a three-chamber cine image.	MVP: Barlow’s phenotype (yes/no); single-leaflet vs bi-leaflet; MVP longitudinal extent; scallops involved in MVP (optional); mitral valve leaflets’ thickness (optional).
		MAD: maximum systolic longitudinal extent in a three-chamber cine image.	MAD: yes/no; longitudinal extent.
		Third-chamber (prolapse volume) [47]	Third-chamber volume (optional) [47]. Regular inclusion of the “third-chamber” volume in the report might improve reproducibility of volumes among different centers and could be useful in patients’ follow-up CMRs. However, it would also increase reporting times, and there are no studies demonstrating an added prognostic value.
MR—qualitative assessment	A stack of three-chamber cine images with high temporal and spatial resolution to fully encompass the mitral valve scallops. 4D-phase-contrast imaging is an alternative in experienced centers.	Visual analysis. By cross-relating short-axis and long-axis cine images in the two orientations, small flails and highly eccentric MR can be singled out.	Single vs multiple jets; jet direction (i.e., central vs eccentric); scallop origin (optional).
MR—quantitative assessment	Indirect method: 2D-phase-contrast through-plane imaging of the thoracic aorta (pulmonary artery) and short-axis stack cine imaging of the LV are required. 4D-phase-contrast imaging is an alternative to the indirect method in experienced centers.	Regurgitant volume (indirect method): LVSV—forward aortic (pulmonary) flow volume. Regurgitant fraction (indirect method): regurgitant volume/LVSV.	Regurgitant volume and regurgitant fraction. Regurgitant fraction should be preferred in patients with impaired ejection fraction. MR is classified as mild or moderate when the regurgitant fraction is <20%, 20–39%, and as severe when the regurgitant fraction and volume are ≥40% and >55–60 mL.
Myocardial fibrosis	LGE imaging according to clinical guidelines [80].	Visual analysis according to clinical guidelines to establish presence, location, and pattern [80]. 5 SD technique to assess LGE extent. Papillary muscles LGE is prone to partial volume artifact and might be assisted by dark-blood or black-blood LGE approaches.	LGE: yes/no; location (AHA segments); pattern (subendocardial vs mid-wall vs subepicardial vs patchy). LGE extent (% of LV mass) is optional.
	T1-mapping imaging including short-axis views and three-chamber long-axis view.	Native T1-mapping values and ECV values of the basal-mid septal segments and of the basal-mid posterior segments.	Septal and posterior native T1-mapping and ECV values (optional).

*AHA* American Heart Association, *CMR* cardiovascular magnetic resonance, *ECV* extracellular volume, *LGE* late gadolinium enhancement, *LV* left ventricle, *LVSV* left ventricular stroke volume, *MAD* mitral annular disjunction, *MR* mitral regurgitation, *MVP* mitral valve prolapse, *SD* standard deviation, *TOE* trans-esophageal echocardiography, *TTE* trans-thoracic echocardiography

*Patients’ selection to CMR:* The clinical selection of patients with MVP who should undergo CMR is debated, and cost-effective studies are lacking. In the context of SCD risk stratification, current guidelines indicate that CMR should be performed in patients with MVP (1) after sustained ventricular arrhythmias before implantable-cardioverter-defibrillator, or (2) in cases of poor acoustic windows when echocardiography does not provide accurate assessment of cardiac chambers’ dimensions or MAD, or (3) in the presence of a history of unexplained syncope and/or non-sustained ventricular tachycardia, and might be considered in patients with MVP and a history of palpitations or T-wave inversion in the inferior leads, repetitive documented polymorphic ventricular arrhythmias, or MAD, enlarged LA or LV ejection fraction <50% at echocardiography [65]. In the context of MR quantification, CMR should be considered in the presence of a discrepancy between patients’ symptoms and MR quantification by echocardiography or in cases of poor acoustic windows or discordant echocardiographic findings [38].

## 5 Gaps in knowledge and future perspectives

The multifaceted prognosis and the poor risk stratification in patients with MVP hold unique research opportunities for the CMR community. However, methodological harmonization is needed, given the lack of a standardized CMR protocol and image interpretation. In particular, there is heterogeneity in the literature on defining and quantifying MAD. Based on the current literature, MAD likely represents a phenotypic signature that accompanies MVP, being associated with the severity of structural and functional MVP abnormalities, and it is unlikely to represent an independent entity. However, further investigations are needed in this direction. The impact of the prolapsing volume (third-chamber) on LV remodeling and the patient’s prognosis remains to be carefully assessed, also taking into account the intriguing hypothesis of the cardiomyopathy-like features observed in MVP. Furthermore, comparative effectiveness research should be encouraged to assess the potential advantage of CMR over the standard-of-care in MVP patients with moderate-to-severe MR in whom CMR-based quantification of LV remodeling and MR severity can emerge as a valid or superior alternative to TTE for clinical decision-making. Finally, a-MVP remains an elusive clinical entity. Large prospective, international, multicenter, and multidisciplinary research should be actively sought to unveil the mechanisms and prognostic markers in patients with an MVP. Furthermore, the impact of surgical or transcatheter intervention on developing life-threatening arrhythmias remains to be defined, given that the current knowledge relies upon retrospective series bias [56], [66], [68]. In this scenario, it is not clear whether CMR could be useful in the follow-up of patients after mitral valve repair or replacement.

## 6 Back to the future: Artificial intelligence at the service of MVP

Machine learning can assist in image acquisition and post-processing of valve and cardiac structures in patients with MVP [85]. However, the role of machine learning in this remains uncharted territory. In a preliminary study, including 429 patients with MVP, a clustering analysis using the Latent Class Model approach was performed to identify echocardiographic patterns. Clusters of patients characterized by LV and LA enlargement and more significant MR at echocardiography were associated with MF at CMR. Clusters were independently associated with cardiovascular outcomes, whereas single clinical or echocardiographic parameters were not. These results emphasize the potential advantage of machine learning in assisting clinical decision-making in patients with MVP and MR [86]. A recent study applied unsupervised machine learning to 474 patients with MVP without moderate-severe MR or LV impairment undergoing CMR. Among the two phenotypic clusters identified, that including patients with more severe mitral valve degeneration, left and right heart chamber remodeling, and MF showed a higher risk of developing an arrhythmic endpoint at follow-up. Notably, demographic and clinical features had negligible contributions in differentiating the two clusters, highlighting the importance of CMR in delineating an MVP phenotype [87].

Furthermore, convolutional neural network algorithms can be developed to assist in the identification and characterization of MVP and MAD in large cohort studies. A multimodal machine learning algorithm could compound clinical history, circulating biomarkers, genetic readouts, ECG, and CMR features to help unveil the key characteristics of an MVP phenotype and thereby identify patients at heightened risk of SCD [86].

## 7 Conclusions

Due to the unparalleled ability to capture the main structural, functional, and tissue abnormalities associated with MVP, CMR is poised as the ideal imaging modality for studying this valvular heart disease. From a clinical standpoint, CMR is already used to complement the standard clinical pathway of patients with MVP by better delineating the extent and severity of structural and functional mitral valve abnormalities, including MR severity, left heart chamber remodeling, and the presence and extent of MF. In perspective, CMR is anticipated to play a key role in better conceptualizing the MVP phenotype, which remains poorly understood. This endeavor would require carrying out a large multicenter international registry incorporating an extensive array of clinical, omics, ECG, and imaging biomarkers ideally empowered by multimodal machine learning algorithms to dissect the mechanisms of an MVP and enable tailored preventive strategies to mitigate the risk of SCD.

## CRediT authorship contribution statement

Bert Vandenberk: Writing—review and editing, Writing—original draft, Investigation, Data curation. Georgios Georgiopoulos: Writing—review and editing, Writing—original draft, Methodology, Investigation, Data curation. Marco Francone: Writing—review and editing, Writing—original draft, Visualization, Validation, Supervision. Amedeo Chiribiri: Writing—review and editing, Validation, Supervision. Steffen E. Petersen: Writing—review and editing, Writing—original draft, Validation, Supervision. Nay Aung: Writing—original draft, Supervision, Investigation. Jan Bogaert: Writing—review and editing, Writing—original draft, Visualization, Validation, Supervision. Tim Leiner: Writing—review and editing, Writing—original draft, Visualization, Validation, Supervision, Conceptualization. Pier-Giorgio Masci: Writing—review and editing, Writing—original draft, Visualization, Validation, Supervision, Project administration, Data curation, Conceptualization. Silvana Di Maio: Writing—review and editing, Writing—original draft, Methodology, Investigation, Data curation. Stefano Figliozzi: Writing—review and editing, Writing—original draft, Supervision, Methodology, Investigation, Data curation, Conceptualization.

## Declaration of Competing Interest

The authors declare that they have no known competing financial interests or personal relationships that could have appeared to influence the work reported in this paper.
